# Global knowledge and attitudes towards mpox (monkeypox) among healthcare workers: a systematic review and meta-analysis

**DOI:** 10.1093/inthealth/ihad094

**Published:** 2023-10-20

**Authors:** Abdolreza Sotoodeh Jahromi, Mohammad Jokar, Nader Sharifi, Sirus Kashkooli, Karamatollah Rahmanian, Vahid Rahmanian

**Affiliations:** Zoonoses Research Center, Jahrom University of Medical Sciences, Jahrom, Iran; Faculty of Veterinary Medicine, Karaj Branch, Islamic Azad University, Karaj, Iran; Department of Public Health, Khomein University of Medical Sciences, Khomein, Iran; Research Center for Social Determinants of Health, Jahrom University of Medical Sciences, Jahrom, Iran; Research Center for Social Determinants of Health, Jahrom University of Medical Sciences, Jahrom, Iran; Department of Public Health, Torbat Jam Faculty of Medical Sciences, Torbat Jam, Iran

**Keywords:** attitudes, awareness, healthcare workers, human monkeypox, knowledge, mpox

## Abstract

**Background:**

The recent increase in human mpox (monkeypox) cases emphasizes the importance of early detection, prompt response and preventive management to control the spread of the disease. Healthcare workers (HCWs) play a crucial role in this process. This study aimed to determine the global knowledge and attitudes towards mpox among HCWs.

**Methods:**

This study searched multiple databases, including Google Scholar, Scopus, PubMed/MEDLINE, Science Direct, Web of Science, Embase, Springer and ProQuest, to locate various publications. The search was limited to English-language articles published between May 2022 (when the increase in mpox incidence was reported) and August 2023. The Joanna Briggs Institute (JBI) quality checklist was utilized to evaluate the quality of the included studies. Data were obtained using a Microsoft Excel spreadsheet and subsequently scrutinized through STATA software, version 14. The heterogeneity of the studies was assessed using the inverse variance and Cochran Q statistics based on the *I*^2^ test statistics. The Dersimonian and Liard random effects models were used where heterogeneity existed. Subgroup analysis and univariate and multivariable metaregression techniques were used to examine the causes of heterogeneity.

**Results:**

A total of 22 studies, including 22 studies for knowledge (27 731 HCWs) and 6 studies for attitudes (14 388 HCWs), were included in the meta-analysis. The pooled estimates for good knowledge and positive attitudes among HCWs were 26.0% (95% confidence interval [CI] 17.8 to 34.2) and 34.6% (95% CI 19.0 to 50.2), respectively. Moreover, the knowledge was 34.8% (95% CI 24.1 to 45.6) among HCWs with <5 y of work experience and 41.6% (95% CI 33.1 to 50) among individuals possessing >5 y of professional background.

**Conclusions:**

Good knowledge of HCWs is at a low level. It is suggested that training sessions should be tailored towards younger HCWs with less healthcare experience. Additionally, it is essential to identify strategies on how to improve the knowledge and attitudes for better practice about the disease in HCWs worldwide.

## Introduction

Mpox (monkeypox) is a zoonotic disease that can be transmitted from animals to humans.^1^ This disease is commonly observed in areas adjacent to tropical rainforests, where animals harbouring the virus can be located. Indications of mpox virus infection have been identified in diverse creatures, including squirrels, Gambian pouched rats, dormice, different monkey species and more.^2,3^

Furthermore, mpox can be transmitted among humans.^3^ Such transmission can occur via interaction with bodily fluids and sores on the skin or internal mucous membranes, such as the oral cavity or throat. Moreover, respiratory droplets and contaminated objects can harbour and disseminate the virus.^4,5^

The symptoms of the disease are similar to those of smallpox, but generally milder.^6^ Despite smallpox having been eradicated in 1980, cases of mpox continue to arise in Central and West African nations.^4,7^ Since May 2022, the disease has been reported in countries that had not previously experienced documented transmission of mpox outside of Africa.^2^

The global outbreak of mpox was declared a public health emergency of international concern on 23 July 2022.^8^ As of 19 June 2023, a total of 87 792 cases and 147 deaths had been reported, including 59 480 cases in the Americas, 25 912 cases in Europe, 1741 cases in Africa, 665 patients in the West Pacific region, 90 cases in the Eastern Mediterranean region and 84 cases in the Southeast Asia region.^8^

Standard protective measures can help prevent the spread of mpox and effective vaccines and treatments are available.^9^ Mpox vaccines are an effective preventive measure that can control the spread of the disease,^10^ with reported protection rates of up to 85%.^11^

A vaccine for mpox (MVA-BN) and a distinct treatment (tecovirimat) were sanctioned for utilization in 2019 and 2022. However, these preventive measures are not yet widely available. The studies suggested that the smallpox vaccination decreases the risk of human mpox, as it is 80.7% efficacious in preventing human mpox and the immunity provided by prior smallpox vaccination is long-lasting.^12–14^

The World Health Organization (WHO) contends that efficient public health monitoring and timely identification via effective medical intervention can avert the transmission of mpox among individuals.^9^ However, this recommendation requires that physicians have sufficient knowledge in diagnosing and treating mpox.^15^

The WHO and national and local health agencies have sought to disseminate information to better inform healthcare workers (HCWs).^16^ Prevention and treatment of infectious diseases often require more than providing information.^17^ Adoption of preventive measures, especially in the context of infectious diseases, is primarily determined by knowledge of the disease, attitudes toward prevention and intention to adopt recommended practices.^18^ Collectively, knowledge, attitudes and practices are known as KAP.

A study in Indonesia showed that the appropriate knowledge of physicians about mpox was 10%, because of a lack of diagnosis of positive samples of mpox until the study was conducted in Indonesia and the lack of education about the disease in the curriculum of medical students.^11^

A study in southern Italy revealed that <66% of hospital HCWs could accurately define mpox. Only 22.8% recognized contact with contaminated objects as a transmission route, with an average mpox knowledge score of 3.4 out of 9. In this study, HCWs with fewer years of working experience and those who had acquired information about mpox from scientific journals had a higher level of knowledge.^19^ Implementing preventative measures, particularly concerning infectious diseases, heavily relies on awareness, the inclination towards prevention and readiness to embrace suggested protocols.^18^

In a study among HCWs in Lebanon, greater knowledge and positive attitudes about mpox were associated with older age and postgraduate education. Physicians demonstrated a notably greater understanding of mpox than other professional groups.^20^

While numerous studies have explored HCWs’ knowledge and attitudes toward mpox,^21–23^ their findings, such as the level of knowledge and attitudes, are inconsistent in some cases. An overall understanding of HCWs’ knowledge and attitudes related to mpox is essential for health system policymakers and stakeholders to design prevention programs. Thus this study aimed to determine the global knowledge and attitudes towards mpox among HCWs.

## Methods

This investigation adhered to the guidelines outlined in the Preferred Reporting Items for Systematic Reviews and Meta-Analyses (PRISMA), which provides 27 standards to ensure precise and transparent reporting.^18,24^ Furthermore, its administrative protocol was recorded in the International Prospective Register of Systematic Reviews (CRD42023439349).

### Search strategy

**Figure 1.**
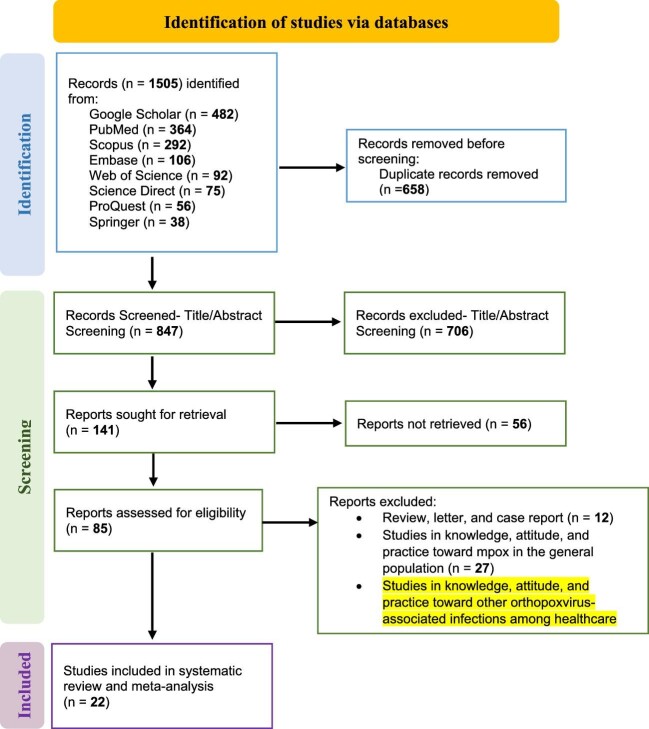
PRISMA flowchart of studies included in this systematic review and meta-analysis.

This study searched multiple databases, including Google Scholar, Scopus, PubMed/MEDLINE, Science Direct, Web of Science, Embase, Springer and ProQuest, to locate various publications. The search was limited to English-language articles published between May 2022 (when the increase in mpox incidence was reported) and August 2023. MeSH phrases were used alone or in combination with other search terms, such as AND, OR and NOT (Supplementary Table 1), to refine the search results and improve their accuracy. To ensure the comprehensiveness of the search, the references of the identified publications were also examined. The process of exploration and the assortment of pertinent articles are depicted in the PRISMA flowchart (Figure 1).

### Inclusion and exclusion criteria

Inclusion criteria were all cross-sectional studies that provided data on knowledge and attitudes regarding mpox in HCWs, as well as studies on KAP in mpox in HCWs, articles published in English and available in full-text format between May 2022 and August 2023 and the reports in which the participants were selected randomly or by census.

Exclusion criteria were articles that failed to meet specific requirements, notably excluding non-observational studies (e.g. short communications) and those targeting populations other than HCWs (e.g. the general population, students).

### Quality assessment (risk of bias)

The Joanna Briggs Institute (JBI) quality rating checklist, specifically designed for cross-sectional studies, was utilized in this analysis to evaluate the quality of the included studies. The JBI checklist comprises nine items that examine the potential for bias in cross-sectional research. These items are categorized into three main groups: study design, sampling strategy and measurement tools. The questions evaluate various aspects of the study, such as the sample's representativeness, appropriate measurement of variables of interest and proper statistical analysis. Each question is rated as yes, no, unclear or not applicable. To ensure the reliability of the quality assessment process, two independent reviewers performed the evaluation using the JBI checklist. The journal's title and the authors’ names were not concealed during the assessment. In case of any discrepancies between the reviewers, a group meeting was held to discuss and resolve them. According to their cumulative score, the studies were classified into three groups: minimal risk of bias (score 8–9), intermediate risk of bias (score 4–7) and substantial risk of bias (score 0–3).^25^

### Data extraction

The data extraction process in this research was executed with meticulous attention to detail, involving several steps. After importing all the selected articles, duplicate entries were removed from EndNote X8 (Clarivate, Philadelphia, PA, USA). The team members individually assessed the remaining publications and examined the titles and abstracts to exclude irrelevant research. The selection criteria were consistent with descriptive and cross-sectional study methods and were based on reports related to the research issue. After identifying relevant articles, the final decision was made through group discussion and the reports were subjected to qualitative assessment and information extraction in the subsequent phases of the research. The data extracted for analysis included several factors, including the author's name, the year of the study, the type of study, sample size, geographic location and participants’ levels of KAP related to mpox.

In this study, knowledge and attitudes about mpox were the following:

- **Knowledge**: Knowledge assessment encompassed information on mpox prevention, diagnosis and treatment. A good level of knowledge means an above-average score.
- **Attitudes:** These statements encompassed opinions regarding the world's ability to control the mpox epidemic and the presence of suitable preventive and control measures. An attitude score above the average level indicates a positive stance towards controlling and managing mpox.

### Statistical analysis

In this meta-analysis, the statistical analysis was conducted using Stata version 14 (StataCorp, College Station, TX, USA). The heterogeneity of the studies was evaluated using the inverse variance and Cochran Q statistics, with the degree of heterogeneity classified as low, moderate or high based on the *I*^2^ test statistics. Heterogeneity was defined as low, moderate or high when the *I*^2^ value was <50%, 50–80% or >80%, respectively.^26,27^ The presence of heterogeneity necessitated the use of Dersimonian and Liard random effects models.^24^

The heterogeneity among the included studies was assessed using several methods, including subgroup analysis, univariate metaregression and multivariable meta-regression techniques. This analysis employed Egger's regression to determine the potential for publication bias. Additionally, the trim-and-fill approach was used to rectify the comprehensive estimate by approximating the number of studies that could have been omitted due to censorship.^28^ The geographic distribution of appropriate knowledge, positive attitude and suitable practice was analysed based on continents and countries using the ArcGIS 10.3 software (Esri, Redlands, CA, USA).

## Results

### Eligibility studies and search results

A comprehensive set of 1505 articles was initially selected from the available databases based on the specific inclusion criteria. However, 658 of these publications were found to have duplicates, leading to their removal from the dataset. The screening process then proceeded to the next step. Upon reviewing the titles and abstracts of the remaining articles, 825 studies were excluded from further consideration for various reasons.

Eventually, after careful assessment, 22 studies were deemed eligible for meta-analysis. Among these, 22 studies pertained to knowledge^11,20,21,29–47^ while 6 focused on attitudes,^20,29,38,42,43,47^ forming the basis for the conclusive analysis (Figure 1).

### Study characteristics

A total of 22 journal articles were selected and satisfied the eligibility criteria for use in this research. Among the 22 studies included, 12 were conducted in Asia, 8 in Africa, 1 in Europe and 1 in the USA. In terms of the types of HCWs, seven studies included physicians,^11,22,29,33,40,42,48^ five studies included medical students,^31,34,38,41,47^ six studies included different healthcare personnel,^20,30,32,37,43,45^ one study included medical and dental practitioners,^35^ one study included medical undergraduates and nursing staff^39^ and one study included dental professionals.^46^

The quality of the chosen studies was evaluated using the JBI checklist. According to the ratings from this checklist, 3 studies were designated as having a minimal risk, while the remaining 19 were labelled as having an intermediate risk (Table 1).

**Table 1.** The included studies in this systematic review and meta-analysis

Study	First author	Year	Study country/region	Study design	Sample size, n	Good level of knowledge, %	Positive attitude, %	Study quality
1	Harapan^11^	2020	Indonesia	Cross-sectional	407	9.3	NR	6
2	Hasan^31^	2023	Bangladesh	Cross-sectional	389	30.59	15.17	7
3	Peng^32^	2023	China	Cross-sectional	459	91.1	NR	7
4	Swed^34^	2023	Algeria	Cross-sectional	96	8.33	NR	7
4	Swed^34^	2023	Egypt	Cross-sectional	617	3.4	NR	7
4	Swed^34^	2023	Iraq	Cross-sectional	296	4.73	NR	7
4	Swed^34^	2023	Jordan	Cross-sectional	382	14.13	NR	7
4	Swed^34^	2023	Kuwait	Cross-sectional	92	5.34	NR	7
4	Swed^34^	2023	Lebanon	Cross-sectional	32	3.13	NR	7
4	Swed^34^	2023	Libya	Cross-sectional	131	5.34	NR	7
4	Swed^34^	2023	Palestine	Cross-sectional	55	14.55	NR	7
4	Swed^34^	2023	Qatar	Cross-sectional	94	22.34	NR	7
4	Swed^34^	2023	Saudi Arabia	Cross-sectional	138	26.81	NR	7
4	Swed^34^	2023	Somalia	Cross-sectional	31	9.68	NR	7
4	Swed^34^	2023	Sudan	Cross-sectional	907	11.8	NR	7
4	Swed^34^	2023	Sultanate of Oman	Cross-sectional	81	12.5	NR	7
4	Swed^34^	2023	Syria	Cross-sectional	1758	7.34	NR	7
4	Swed^34^	2023	United Arab Emirates	Cross-sectional	50	16	NR	7
4	Swed^34^	2023	Yemen	Cross-sectional	1183	10.65	NR	7
5	Hidar Alibrahim^33^	2023	Syria	Cross-sectional	1257	0.23	NR	7
6	Gonzales-Zamora^35^	2023	USA	Cross-sectional	463	46.65	NR	6
7	Alshahrani^36^	2022	Saudi Arabia	Cross-sectional	314	28.02	NR	7
8	Joseph^37^	2023	India	Cross-sectional	424	64.9	NR	7
9	Harapan^38^	2020	Indonesia	Cross-sectional	432	36.5	NR	7
10	Sobaikhi^39^	2023	Saudi Arabia	Cross-sectional	398	44.1	NR	8
11	Kumar Das^45^	2023	Nepal	Cross-sectional	205	39.6	48.3	8
12	Ahmed^41^	2022	India	Cross-sectional	340	17.05	NR	6
13	Iwuafor^42^	2022	Nigeria	Cross-sectional	164	23.2	NR	4
14	Bhadra^43^	2022	India	Cross-sectional	152	43.42	NR	5
15	Malaeb^20^	2023	Lebanon	Cross-sectional	646	33.7	30.7	7
16	Alshahrani^21^	2022	Saudi Arabia	Cross-sectional	398	55	NR	7
17	Sahin^44^	2023	Turkey	Cross-sectional	283	32.5	41.7	7
18	Kumar^40^	2022	Pakistan	Cross-sectional	946	6.3	20.5	8
19	Lounis^46^	2022	Algeria	Cross-sectional	111	63.06	NR	6
20	Gebreal^47^	2023	Egypt	Cross-sectional	1740	35.49	NR	4
21	Kaur^48^	2022	India	Cross-sectional	410	28	NR	6
22	ElHafeez^49^	2023	NR	Cross-sectional	11 919	55.3	51.7	6

NR: no report.

### Pooled good knowledge of mpox

A comprehensive analysis was conducted on 22 studies involving 27 731 HCWs to assess their level of knowledge regarding mpox. The pooled estimation of the proportion of good knowledge was determined using a random effects model, considering the presence of heterogeneity (I–V heterogeneity).

**Figure 2.**
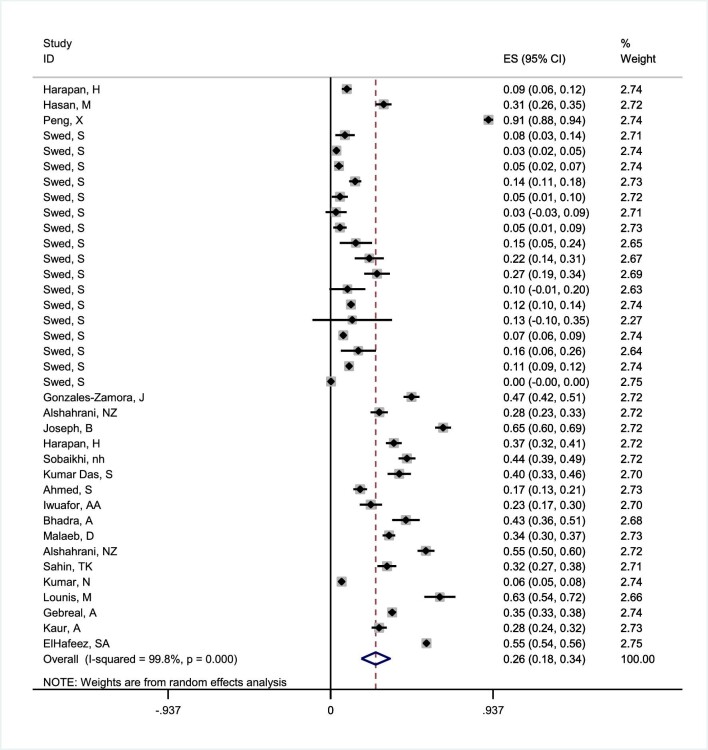
Forest plot of I–V heterogeneity random effects meta-analysis for good knowledge of mpox among HCWs.

The overall knowledge of mpox among HCWs was 26.0% (95% CI 17.8 to 34.2) (Figure 2). However, there was a significant level of heterogeneity (*I*^2^=99.8%, Q statistic=21105.71, df=36, p<0.0001, τ^2^=0.0645) among the studies.

We conducted a thorough sensitivity analysis using the one-by-one studies method, and the results revealed that no single study had a significant impact on the proportion of good knowledge. As a result, we did not identify any influential studies in this respect (Supplementary Figure 1).

Univariate and multivariate metaregression were utilized to pinpoint potential origins of the heterogeneity observed within the study. The results of multiple regression showed that none of the variables, including study quality, continent, country, year of study or sample size, are likely sources of heterogeneity (p>0.05). However, univariate metaregression indicated that continent (β=−0.022, p=0.032) may be a potential source of heterogeneity in studies related to knowledge (Table 2).

**Table 2.** Univariate and multivariable metaregression to find possible causes of heterogeneity among studies included in the meta-analysis

		Univariate		Multivariable	
Type	Possible cause of heterogeneity	Coefficient (95% CI)	p-Value	Coefficient (95% CI)	p-Value
Knowledge	Quality of study	−0.0198 (−0.0984, 0.0586)	0.620	−0.0075 (−0.0999, 0.0848)	0.869
	Continent	−0.0226 (−0.1565, −0.1111)	0.033	−0.0086 (−0.1273, 0.1101)	0.448
	Country	0.0011 (−0.0106, 0.0129)	0.843	−0.0002 (−0.0114, 0.0109)	0.967
	Year	−0.0161 (−0.1061, 0.0731)	0.713	−0.0191 (−0.1244, 0.0862)	0.714
	Sample size	0.00002 (−0.00001, 0.0000569)	0.197	0.00002 (−0.00001, 0.00006)	0.277
Attitude	Quality of study	−0.0657 (−0.2155, 0.0839)	0.389	0.0976 (−2.725, 2.920)	0.736
	Continent	0.1342 (−0.1105, 0.3789)	0.283	0.1001 (−1.6986, 1.898)	0.608
	Country	0.0325 (−0.0258, 0.0908)	0.275	0.0045 (−0.9348, 0.9439)	0.961
	Year	0.1690 (−0.0911, 0.4293)	0.203	0.1420 (−0.3703, 0.6544)	0.443
	Sample size	0.000022 (0.000020, 0.000025)	<0.001	0.00001 (−0.00002, 0.00005)	0.359

**Figure 3.**
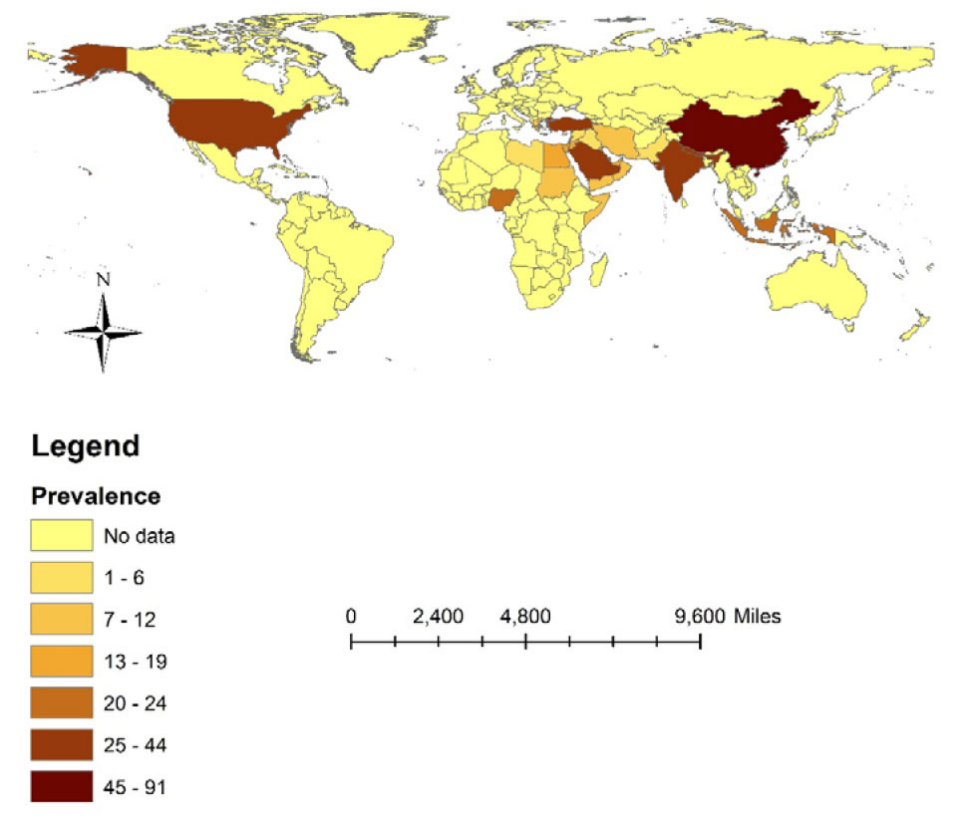
Percentage of good knowledge of mpox among HCWs by country.

Based on subgroup analysis, the level of knowledge regarding mpox among healthcare personnel in different continents was as follows: 46.7% (95% CI 42.1 to 51.2) in the USA, 32.5% (95% CI 27.0 to 38.0) in Europe, 26.1% (95% CI 18.1 to 34) in Asia and 17.1% (95% CI 9.0 to 25.1) in Africa (Table 3, Figure 3). Moreover, the knowledge was 34.8% (95% CI 24.1 to 45.6) among individuals with <5 y of work experience and 41.6% (95% CI 33.1 to 50) among individuals with >5 y of experience. Additionally, the knowledge was 27.8% (95% CI 22.0 to 35.5) in personnel <30 y of age and 44.4% (95% CI 32.4 to 56.4) in individuals >30 y of age. There was no significant difference in knowledge between men and women (34.5% [95% CI 26.1 to 42.8] vs 35.8% [95% CI 28.8 to 42.9]) (Table 3).

**Table 3.** The results of subgroup analysis based on country and continent for KAP for COVID-19 in chronic disease patients

	Grouping					Heterogeneity			
Type			Studies, n	Individuals examined, n	Overall frequency, % (95% CI)	χ^2^	p-Value	*I* ^2^, %	τ^2^
Knowledge	Continent	Africa	8	2520	17.1 (9.0 to 25.1)	218.92	<0.001	97.3	0.0107
		Asia	27	24 874	26.1 (18.1 to 34)	8409.40	<0.001	99.7	0.0434
		Europe	1	283	32.5 (27.0 to 38.0)	NA	NA	NA	NA
		Americas	1	50	46.7 (42.1 to 51.2)	NA	NA	NA	NA
	Work Experience	<5 y	11	3815	34.8 (24.1 to 45.6)	1236.46	<0.001	99.2	0.0322
		≥5 y	10	1755	41.6 (33.1 to 50)	117.09	<0.001	92.3	0.0165
	Sex	Male	14	5188	34.5 (26.1 to 42.8)	1703.74	<0.001	99.2	0.0241
		Female	14	6716	35.8 (28.8 to 42.9)	1962.97	<0.001	99.3	0.0169
	Age group	<30 y	11	8794	27.8 (22.0 to 35.5)	0.0083	<0.001	99.2	0.0083
		≥30 y	9	1839	44.4 (32.4 to 56.4)	218.10	<0.001	96.3	0.0321
Attitude	Continent	Asia	5	14 105	28.2 (18.0 to 38.5)	92.78	<0.001	96.8	0.0105
		Europe	1	283	41.7 (36.0 to 47.4)	NA	NA	NA	NA
	Work Experience	<5 y	3	504	57.4 (27.4 to 87.7)	111.76	<0.001	98.2	0.0673
		≥5 y	3	373	62.2 (33.2 to 91.1)	73.83	<0.001	97.3	0.0634
	Sex	Male	4	847	48.6 (12.0 to 85.1)	446.53	<0.001	99.3	0.1378
		Female	4	976	50.6 (17.4 to 83.9)	397.54	<0.001	99.2	0.1136
	Age group	<30 y	4	1334	45.5 (14.0 to 76.9)	375.67	<0.001	99.2	0.1016
		≥30 y	4	489	54.2 (22.5 to 85.8)	207.81	<0.001	98.6	0.1024

NA: not applicable.

### Pooled good attitudes towards mpox

**Figure 4.**
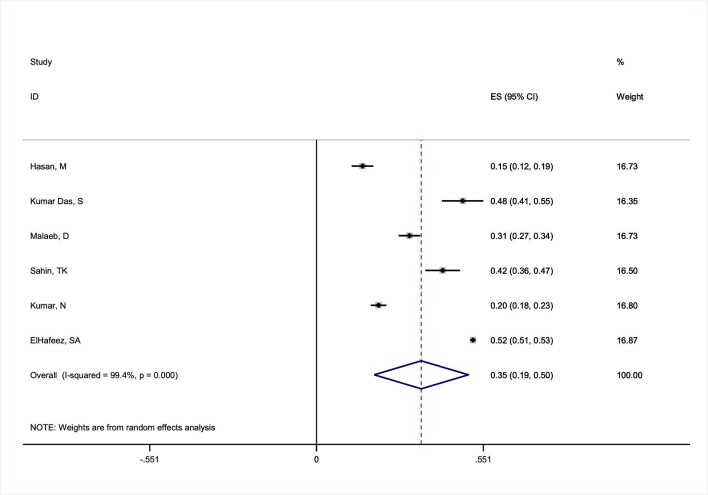
Forest plot of I–V heterogeneity random effects meta-analysis for a good attitude of mpox among HCWs.

A total of six studies with 14 388 participants were assessed for the attitude analysis. According to the random effects model with I–V heterogeneity, the percentage of HCWs with positive attitudes was 34.6% (95% CI 19.0 to 50.2) (Figure 4). However, there was a significant level of heterogeneity (*I*^2^=99.4%, Q statistic=892.94, df=6, p<0.0001, τ^2^=0.0375) among the studies. We conducted a thorough sensitivity analysis by removing studies one by one and the results revealed that no single study had a significant impact on the proportion of good knowledge. As a result, we did not identify any influential studies in this respect based on the analysis (Supplementary Figure 2).

The outcomes of the univariate metaregression revealed that sample size (β=0.000022, p<0.001) could account for the heterogeneity. Conversely, the multivariable metaregression findings indicated that none of the variables, including study quality, continent, country, year of study or sample size, appear to be probable sources of heterogeneity (p>0.05) (Table 2).

**Figure 5.**
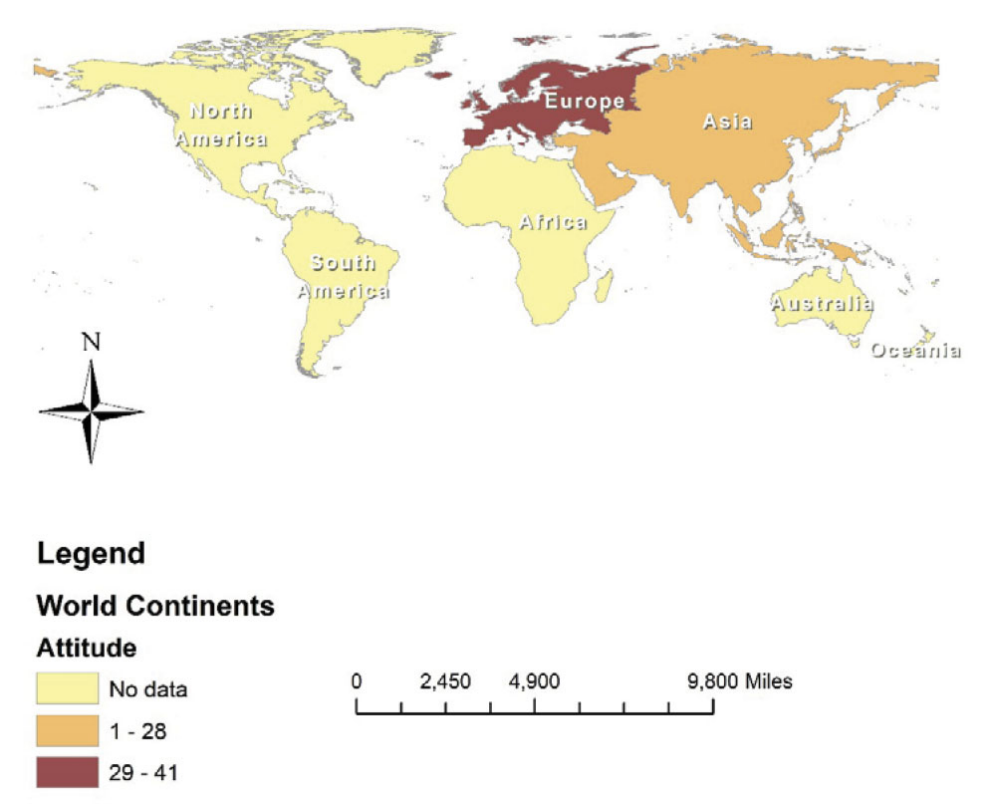
Percentage of the good attitude of mpox among HCWs by continent.

The findings from the subgroup analysis demonstrated that the favourable attitude towards mpox among healthcare personnel in Europe stood at 41.7% (95% CI 36.0 to 47.4), while in Asia it was 28.2% (95% CI 18.0 to 38.5) (Figure 5 and Table 3). Furthermore, the positive attitude among personnel <30 y of age was 45.5% (95% CI 14.0 to 76.9), while it was 54.2% (95% CI 22.5–85.8) in those ≥30 y of age. The attitude among personnel with <5 y of work experience was 57.4% (95% CI 27.4–87.7), while it was 62.2% (95% CI 33.2 to 91.1) in those with ≥5 y of experience. There was no difference in attitude between men and women )48.6% vs 50.6%) (Table 3).

### Publication bias

**Figure 6.**
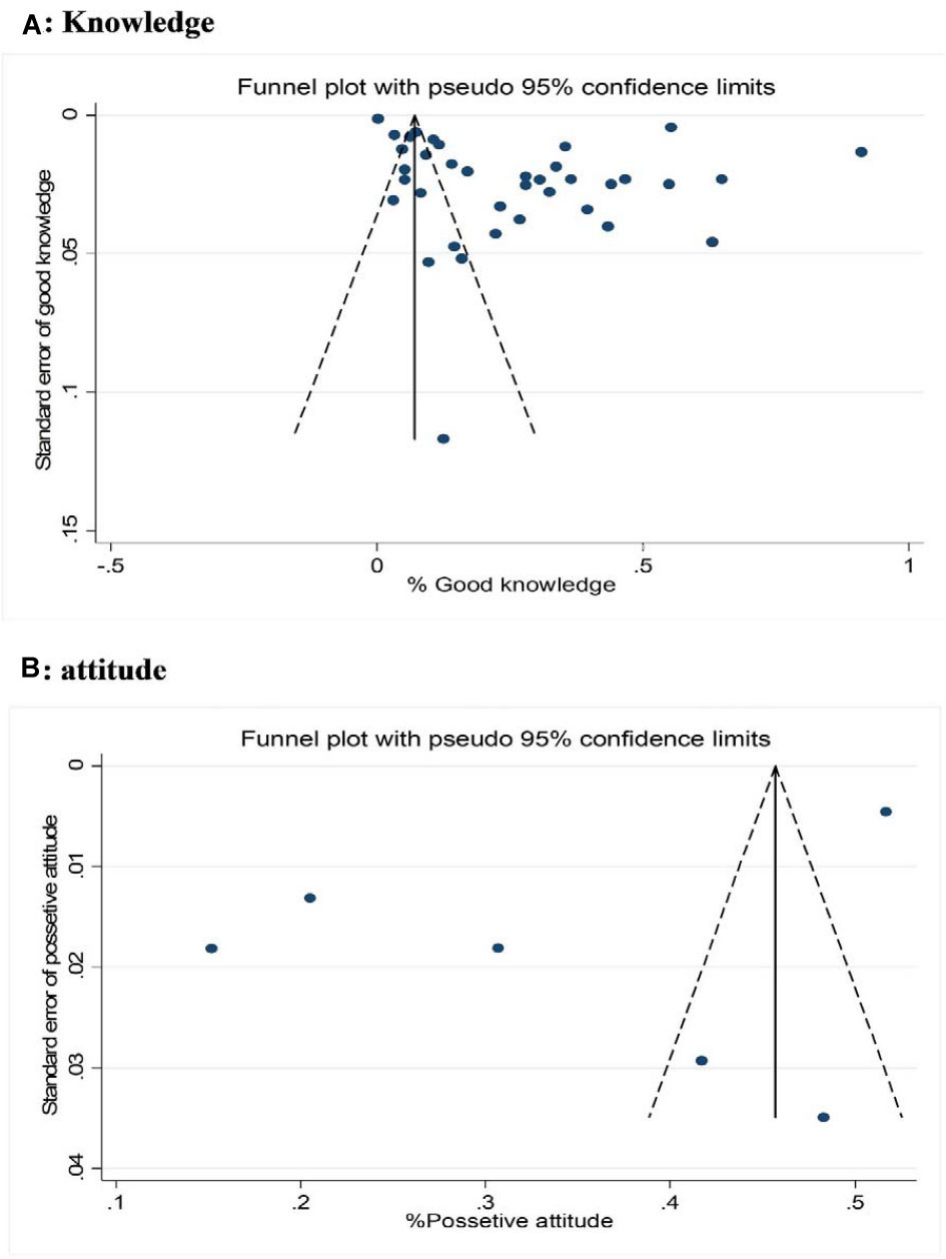
Funnel plot with pseudo 95% CIs for detection of publication bias among included studies.

The outcomes of Egger's regression test and the irregularity in the funnel plot demonstrated notable publication bias within the studies encompassing knowledge that were incorporated in this meta-analysis (bias 12.94 [95% CI 4.28 to 21.60], p=0.005) (see Figure 6A). A non-parametric trim-and-fill model was applied to rectify this bias, revealing an estimation of seven hypothetical studies regarding knowledge of mpox among HCWs that could be absent from the meta-analysis. Utilizing this technique, the adjusted pooled proportion of good knowledge, determined through the random effects model, was appraised at 31.1% (95% CI 20.2 to 42.1).

Additionally, Egger's test indicated the absence of significant publication bias within the studies related to attitudes encompassed in this meta-analysis (bias −12.96 [95% CI −32.44 to 6.51], p=0.138). The symmetric distribution of studies in the funnel plot analysis (Figure 6B) did not confirm the presence of publication bias, further supporting the robustness of the study's findings.

## Discussion

The study revealed that the overall knowledge of mpox among HCWs was 26.0%, indicating that many HCWs have limited knowledge of this infectious disease. This finding is concerning as it highlights potential gaps in the knowledge necessary for the early detection, diagnosis and management of mpox cases. Inadequate knowledge can lead to delays in identification and response, potentially contributing to the spread of the disease and compromising patient care. Of course, the findings of some studies are somewhat different from these general results. In the study of Peng et al. in China,^30^ Lounis et al. in Algeria^44^ and Joseph et al. in India,^35^ a high level of good knowledge about mpox was obtained. It seems that in some countries, due to the experience of coronavirus disease 2019 (COVID-19), programs have been implemented to increase the knowledge of HCWs about mpox. In contrast, the lowest level of good knowledge of HCWs was found in Syria (0.23), Lebanon (3.13), Egypt (3.4), Iraq (4.73), Libya (5.34) and Pakistan (6.3).^31,32,38^

The results of studies on the general population's knowledge also show a low level of good knowledge about mpox.^49–51^ The remarkable thing is that there is little difference between the general public's knowledge and that of HCWs. It should be noted that with the reduction of importance of some diseases, such as smallpox, teaching these diseases is not considered in the educational resources of medical sciences. These cases show the need to revise the teaching resources along with the implementation of health education programs by improving knowledge about such diseases.

Furthermore, the study found that 34.6% of HCWs had positive attitudes towards mpox. Positive attitudes are crucial for effective disease control and prevention efforts, as they drive the willingness of HCWs to engage in surveillance, reporting and implementation of preventive measures.

Although it is necessary to have a good attitude and knowledge, it seems that the fear created in society and HCWs regarding the creation of a new deadly pandemic after the experience of COVID-19 has created a higher good attitude. However, there is not enough knowledge about the different aspects of the disease. The results of other studies also indicate the existence of a feeling of relative concern about this disease at the community level.^52,53^

In many of the reviewed articles, an exact measure of the level of good attitude is not provided, and more studies are needed in this field. Rational action in preventing mpox will require knowledge and sensitivity in society and HCWs. This is of much greater importance in the case of HCWs, because they are directly involved with the community's health.

## Strengths and limitations

This study has several limitations. First, its scope was confined to English-language articles exclusively, potentially resulting in language bias and the exclusion of pertinent non-English studies. This could compromise the comprehensiveness and applicability of the findings, especially when viewed from a global perspective. Second, an identified challenge was the potential presence of publication bias in evaluating knowledge. This could potentially inflate the prevalence estimation of strong knowledge among HCWs. Although the study endeavoured to address this bias by implementing the trim-and-fill method, the exact impact of omitted studies on the outcomes remains uncertain. Lastly, the study undertook a geographical analysis using ArcGIS software to examine knowledge and attitudes across continents and countries. Yet this geographical assessment might oversimplify intricate regional disparities encompassing healthcare systems, educational attainment and socio-economic variables that could influence the findings.

Notwithstanding these limitations, the study boasts noteworthy strengths. HCWs are pivotal in patient education, infection control and the overall provision of healthcare services. Consequently, the study's revelations concerning the knowledge and attitudes of HCWs can yield direct benefits in enhancing the quality of patient care. By ensuring the accurate dissemination of information and the implementation of effective infection prevention measures, this research holds the potential to contribute significantly to healthcare outcomes.

## Conclusions

The study results showed that good knowledge of HCWs is at a low level. Due to the direct involvement of HCWs with the health of the community and to create the necessary preparation in these people for the emergency of mpox, it is suggested that training sessions should be tailored towards younger HCWs with less healthcare experience. These sessions can be led by older people with more experience and from countries/regions that were found to have the highest levels of good knowledge and attitudes.

Future studies can contribute to bridging the identified gaps due to language and the discrepancies not reported here within regions, particularly regarding the weaknesses of our analysis. Other studies could review the correlation between knowledge and attitudes and outcomes (standardized prevention measures, vaccination and treatment) for mpox.

## Supplementary Material

ihad094_Supplemental_Files

## Data Availability

The data supporting the findings are presented in the article.
