# Supplementary material for: Global knowledge and attitudes towards mpox (monkeypox) among healthcare workers: a systematic review and meta-analysis
Source: Int Health. 2023 Oct 20;16(5):487–98. doi: 10.1093/inthealth/ihad094 (PMC11375569; doi:10.1093/inthealth/ihad094)
Supplement: ihad094_Supplemental_Files [file ihad094_supplemental_files.zip › Supplementary Figures.docx]

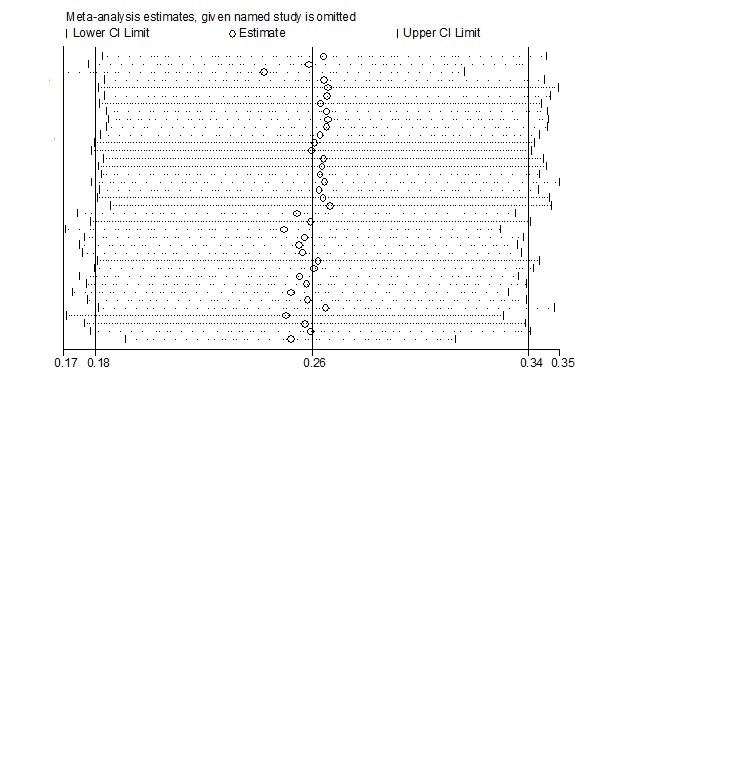


Supplementary Figure1. Sensitivity analysis of knowledge by removing one-by-one studies

Supplementary Figure2. Sensitivity analysis of attitude by removing one-by-one studies
