# Supplementary material for: Global knowledge and attitudes towards mpox (monkeypox) among healthcare workers: a systematic review and meta-analysis
Source: Int Health. 2023 Oct 20;16(5):487–98. doi: 10.1093/inthealth/ihad094 (PMC11375569; doi:10.1093/inthealth/ihad094)
Supplement: ihad094_Supplemental_Files [file ihad094_supplemental_files.zip › supplementary table 1.docx]

Table S1: Systematic Search Strategy for Identifying Studies Assessing Knowledge, Attitudes, and Practices (KAP) towards Mpox (monkeypox) among Health Care Workers.

| **Databases** | **Search terms** |
| --- | --- |
| Google Scholar, Scopus, PubMed/MEDLINE, Science Direct, Web of Science, EMBASE, Springer, and ProQuest | ("Monkeypox" [MESH] OR Mpox [MESH] OR "Orthopoxvirus Infections" [MESH]) AND ("Health Personnel" [MESH] OR "Healthcare Workers" [MESH] OR "Medical Staff" [MESH]) AND ("Knowledge" [MESH] OR "Attitude" [MESH] OR "Practice" [MESH] OR "Behavior" [MESH]) AND "Prevention and Control" [MESH] OR "Disease Outbreaks" [MESH]) NOT ("Animals" [MESH] OR "Animal Diseases" [MESH]) |
| **Number of paper identified** | **Results in each database** |
| 1505 Journal papers | Google Scholar= 482 papers  PubMed= 364 papers  Scopus= 292 papers  Embase= 106 papers  Web of Science= 92 papers  Science Direct= 75 papers  ProQuest= 56 papers  springer= 38 papers |
